# GatewayNet: a form of sequential rule mining

**DOI:** 10.1186/s12911-019-0810-3

**Published:** 2019-04-23

**Authors:** Phillip C. S. R. Kilgore, Nadejda Korneeva, Thomas C. Arnold, Marjan Trutschl, Urška Cvek

**Affiliations:** 1 Department of Computer Science, LSU Shreveport, 1 University Place, Shreveport, 71115 USA; 2 Department of Pharmacology, Toxicology and Neuroscience, LSU Health Shreveport, 1501 Kings Highway, Shreveport, 71103 USA; 3 Department of Emergency Medicine, LSU Health Shreveport, 1501 Kings Highway, Shreveport, 71103 USA; 4 Center for Molecular and Tumor Virology, LSU Health Shreveport, 1501 Kings Highway, Shreveport, 71103 USA

**Keywords:** Initiation rules, Gateway hypothesis, Association rule mining, Causal network, Structure learning

## Abstract

**Background:**

The gateway hypothesis (and particularly the prediction of developmental stages in drug abuse) has been a subject of protracted debate since the 1970s. Extensive research has gone into this subject, but has yielded contradictory findings. We propose an algorithm for detecting both association and causation relationships given a discrete sequence of events, which we believe will be useful in addressing the validity of the gateway hypothesis.

To assess the gateway hypothesis, we developed the GatewayNet algorithm, a refinement of sequential rule mining called initiation rule mining. After a brief mathematical definition, we describe how to perform initiation rule mining and how to infer causal relationships from its rules (“gateway rules”).

We tested GatewayNet against data for which relationships were known. After constructing a transaction database using a first-order Markov chain, we mined it to produce a gateway network. We then discuss various incarnations of the gateway network.

We then evaluated the performance of GatewayNet on urine drug screening data collected from the emergency department at LSU Health Sciences Center in Shreveport. A de-identified database of urine drug screenings ordered by the department between August 1998 and June 2011 was collected and then restricted to patients having at least one screening succeeding their first positive drug screening result.

**Results:**

In the synthetic data, a chain of gateway rules was found in the network which demonstrated causation. We did not find any evidence of gateway rules in the empirical data, but we were able to isolate two documented transitions into benzodiazepine use.

**Conclusions:**

We conclude that GatewayNet may show promise not only for substance use data, but other data involving sequences of events. We also express future goals for GatewayNet, including optimizing it for speed.

**Electronic supplementary material:**

The online version of this article (10.1186/s12911-019-0810-3) contains supplementary material, which is available to authorized users.

## Background

The *Gateway hypothesis* (also *gateway theory* or *stepping-stone theory*) is the assertion that the use of certain psychoactive drugs (e.g., tobacco, alcohol, or cannabis) increases the likelihood that other drugs will later be used. It is commonly interpreted to mean that usage of one drug will encourage the initiation (or first usage) of new substances, and the first drug is therefore said to be called a *gateway drug*. Another prediction that has been associated with the gateway hypothesis is that initiation for specific drugs develops in stages.

### The gateway hypothesis

This hypothesis is controversial amongst substance abuse experts, as many studies with conflicting results have been released since intense interest beginning in the 1970s. For instance, Kandel originally predicted a chain of drug use progression from tobacco and alcohol to cannabis, then to LSD, amphetamines, or heroin. She posits that this association is bidirectional and that a similar sequence will occur for regression in drug use [1]. In 1984, a follow-up was performed to address the fact that detailed monitoring of adolescents into young adulthood, suggesting that initiation risk may be partially conditional on age and that risk progresses in stages [2]. At the height of the crack cocaine epidemic, Kandel and Yamaguchi reformed their model to account for its sudden appearance and found that a) cocaine precedes crack cocaine, and b) models using cocaine or crack cocaine exclusively had a poorer fit than those containing both [3].

O’Donnell and Clayton directly claimed a causal connection between marijuana and heroin use [4]. To support this, they note that marijuana and heroin are statistically associated, that marijuana precedes heroin use, and that this association is not spurious. O’Donnell and Clayton alleged that a large cohort of sociologists were skeptical of the gateway hypothesis at the time, and they argued that marijuana causes heroin use according to how sociologists understand causation [4].

Early criticism of causal predictions of the gateway hypothesis takes two major forms: that the evidence does not support the assertion or that the assertion is structurally flawed. In an attempt to replicate Kandel’s work, Baumrind obtained a different pathway which implicated that tobacco succeeded cannabis (though both found that the use of socially-accepted substances precedes that of the unacceptable), noting that drug initiation order may be influenced by sociocultural aspects [5].

An additional form of criticism arose in the way that the conclusion itself was being formulated. In an article warning against drawing false conclusions of causation, Baumrind cites O’Donnell and Clayton as an exemplar of this [6]; she later comments that Guttman scales cannot be extrapolated into a sequence of development stages as was done in Kandel’s work [5]. Vanyukov et al. argue that the gateway hypothesis may lack falsifiability and that the concept itself is vague [7].

Nonetheless, contemporary support of the gateway hypothesis is mixed. It is known that rats exposed to *Δ*9-tetrahydrocannabinol (THC, the primary active compound in cannabis) will increase self-administration of nicotine, heroin, and morphine [8–10], showing that cannabis can operate as a gateway drug outside of any particular cultural context. Conversely, it has been argued that the apparent progression is one of several, and that common liability to addiction may be enough to explain patterns in substance use [7]. One longitudinal study of New Zealand children concluded that although there was strong association with a diverse use of other drugs and that this may support a causal model, the underlying causal mechanisms are not well understood [11].

There are two major approaches involving longitudinal data used to assess drug use in human subjects: through self-reporting and through urine drug screening (UDS). In self-reporting studies, subjects are asked to inform investigators about their drug history. This method frequently tracks subjects from adolescence into adulthood to determine both trends in usage and initiation. However, it may be influenced by response bias common to interviews and surveys [12, 13].

UDS detects metabolites associated with certain drugs use (usually via a panel assay). This offers a major advantage over self-reporting: it is possible to collect information that would otherwise be withheld in a self-reporting study. It also becomes possible to collect data from subjects who are unable to participate in interviews, such as infants (who are unlikely to consciously participate in drug use, but which may reveal drug use by parents).

The main disadvantage of this method is false positive results arising from misidentification of metabolites in urine. For instance, it is known that quinolone antimicrobials can create false positives for opiate presence [14, 15]. Several forms of medication (both prescribed and over-the-counter) are known to trigger false positives in drug tests; ibuprofen, a common analgesic, may trigger false positives for phencyclidine (PCP), cannabinoids, and barbiturates in some screening panels [15].

### Previous approaches

The goal of GatewayNet is to predict initiation events and select those relationships which may be causal; therefore, it is important to consider past approaches to this problem. It should be noted that the causation referred to here is not deterministic causation: observation does not support the idea that a gateway drug is always followed by its target. Instead, the idea of *probabilistic causation* (i.e., event *a* is likely to cause *b*) is considered [16].

1 $$ p\left(b | do(a)\right) > p\left(b | do(\neg a)\right)  $$

Statistical treatment of this problem has been attempted in the literature. A simple method uses a linear probability model [11, 17], such as the one suggested by Beenstock and Rahav to predict how cigarettes influenced cannabis use Eq. 2, where *S*_*nt*_ and *C*_*nt*_ are indicators of cigarettes and cannabis respectively by sample *n* at time *t*, *X* is a vector of personality characteristics, *D*_*y*_ is the birth cohort for year *y*, and *u*_*nt*_ accounts for unobserved error. The gateway hypothesis predicts that if *C* is a gateway into *S*, then *β*>0 [17].

2 $$ S_{nt} = \alpha X_{nt} + \beta C_{n(t-1)} + \gamma_{y} D_{y} + u_{nt}  $$

Hazard analysis has also been used to assess this problem [17]. In relation to the gateway hypothesis, hazard analysis attempts to ascertain the risk of initiating the use of another drug. Recently, latent transition analysis has been used to assess gateway relationships [18].

Bayesian inference is often used to assess claims of causation. For instance, a Bayesian method was applied to assess data from Norwegian young adults and yielded the conclusion that proneness and accessibility are important contributing factors to hard drug use [19]. Another potential avenue might be in the form of a Bayesian Belief Network (BBN), a directed acyclic graph describing the probability of condition *b* occurring given condition *a* [20]; however, the literature does not record such an application of BBNs to the gateway hypothesis.

### Association rule mining

Association Rule Mining (ARM) is a well-known method where a set of items called a *transaction* can be mined to produce *association rules* of the form *a*→*b*, which is a prediction that when *a* is present, *b* will co-occur. A related strategy, known as *sequential rule mining* (SRM), can be used to predict that *a* will *precedeb* in sequence. Algorithms which use SRM include the Co-occurrence Maps with Sequence PAttern Mining using Equivalent class (CM-SPADE) [21], Sequential PAttern Mining (SPAM) [22], and Closed Sequential Patterns (ClaSP) [23] algorithms.

Sequential rule mining is applicable to a problem such as the Gateway Hypothesis because the latter predicts a causal relationship; if *a* causes *b*, then it is necessary for *a* to precede *b*. Causation also implies that the first instance of *b* will not precede the first instance of *a*.

We claim three contributions to the literature: i) the application of sequential rule mining to the assessment of the Gateway hypothesis, ii) the use of these rules to construct a *gateway network* describing interaction between, and iii) the introduction of the *certainty* measure.

## Implementation

To better understand the extent to which the Gateway Hypothesis manifests itself in drug use trends, we developed GatewayNet, an algorithm that constructs a directed, weighted graph of drug initiation events derived from a form of association rule mining. We then performed an evaluation against two data sets: a synthetic data set, and an empirical data set derived from UDS data.

### Mathematical model

In the following paragraphs, the mathematical basis for GatewayNet (and in particular, initiation rule mining) are described. How this model is defined is critical to interpreting GatewayNet’s results, so it is described in detail here.

**Precedence Relations** Let *E* denote a set of events, $S : t \in \mathbb {Z}^{+} \mapsto E$ denote a sequence of events called the *history* such that *S*_*t*_⊆*E* is the set of events occurring at some time *t*, *a*⊆*E*, and *b*⊆*e*. The predicate *a*≺*b* means “*a* precedes *b*” and is defined in Eq. 3.

3 $$ a \prec b \equiv \exists t \in \mathbb{Z}^{+} : a \subseteq S_{t} \wedge b \subseteq S_{t+1}  $$

It should be noted that *a*≺*a* may yield true under this definition. The operand *a* is called the antecedent, while *b* is called the subsequent.

**Initiation Relations** Let $S^{a}_{b} = S_{a} \cup... \cup S_{b}$. For brevity, $S_{t}^{*} = S^{1}_{t}$ and $S^{*} = S^{*}_{|S|}$. The predicate *a*⊆*b* means “*a* initiates *b*” (an instance thereof being called an *initiation rule*) and is defined in Eq. 4. An initiation rule *a*⊆*b* has a degree which is the maximum between the number of elements in *a* and the number of elements in *b* Eq. 5.

4 $$ \begin{array}{ll} a \rightarrow b & \equiv \exists t : a \prec b \wedge b \not\subseteq Sn{t}\\ & \equiv \bigvee^{|S|-1}_{t=1} a \subseteq S^{*}_{t} \wedge b \subseteq S_{t+1} \in b \not\subseteq S^{*}_{t} \end{array}  $$

5 $$ \text{deg} \left(a \rightarrow b\right) \equiv \text{max}\left(|a|,|b|\right)  $$

Note that (unlike precedence relations) the initiation relation *a*→*a* is universally false. This relation can be further generalize d into windowed initiation. Let $z \in \mathbb {Z}+$, *z*^′^=*z*−1, and $a \overset {z}{\rightarrow } b$ denote an initiation rule within window *z*. In this generalization, only the most recent *z* time points are searched for the antecedent in every time-step. Because $a \overset {0}{\rightarrow } b$ is trivially false according to Eq. 6, it has been redefined Eq. 7.

6 $$ a \overset{z}{\rightarrow} b \equiv \bigvee^{|S|-1}_{t=1} a \subseteq S^{t-z^{\prime}}_{t} \wedge b \subseteq S_{t+1} \wedge b \not\subseteq S^{t-z^{\prime}}_{t}  $$

7 $$ a \overset{0}{\rightarrow} b \equiv a \rightarrow b  $$

The purpose behind this generalization is to account for large gaps of time between two events. For instance, if an event occurs in *S*_1_ and is not recorded thereafter, can it be said to be associated with an event a time *τ*? With windowed initiation $a \overset {\tau -}{\rightarrow } b$, this question can be answered.

It is trivial to show that the set of initiation *z*-windowed rules is a subset of the set of all initiation rules: the set of rules $a \overset {0}{\rightarrow } b$ are equivalent to *a*→*b* and is vacuously a subset, and because $S^{t-z^{\prime }}_{t} \subseteq S^{*}_{t}$ by definition, all initiation rules for *z*>0 are also initiation rules. Thus, the rule $a \overset {z}{\rightarrow } b$ implies *a*→*b*.

### Initiation rule mining

We elicit initiation rules de novo using a method we call *initiation rule mining* (IRM). IRM is similar in design to ARM: candidate rules are proposed, then based off of their support in a transaction database, are assessed for their validity. The primary difference is that rather than looking within the same transaction, IRM mines rules by looking between different transactions contained in a single history.

Let *T* represent a set of histories (the transaction database) and *S*∈*T*. One possible incarnation of *T* (the incarnation used by GatewayNet) is illustrated in Table 1. Each record within the table is a triple (*i*,*t*,*S*_*t*_), such that *T*_*i*(*t*)_=*S*_*t*_.

**Table 1:** A sample transaction database

ID	Time	Itemset	ID	Time	Itemset
1	0	{*I*_1_,*I*_2_}	4	0	{*I*_1_}
1	1	{*I*_3_}	4	2	{*I*_2_,*I*_3_}
1	4	{*I*_2_,*I*_3_}	4	3	{*I*_1_,*I*_2_}
2	0	{*I*_2_}	5	0	{*I*_1_}
3	2	{*I*_1_}	5	1	{*I*_1_,*I*_2_}
3	5	{*I*_2_}	5	4	{*I*_1_,*I*_2_,*I*_3_}

Criteria must exist for candidate rules to be accepted or rejected, and several are traditionally used in ARM that apply here. Count Eq. 8 and support (Eqs. 9 and 10) are perhaps the most basic and may be used to filter out rules which run the risk of being statistically invalid [20, 24]; however, high limits may preclude many relationships from being discovered. Confidence is a measure of how likely the rule occurs when its antecedent occurs Eq. 11 and may be a more suitable measure for this purpose. Lift Eq. 12 is a measure of interest which considers the case where *a* and *b* are independent [20]. Finally, conviction is the frequency that the rule makes an incorrect prediction Eq. 13 [20]. Thresholds for inclusion are expressed as *l*_*count*_, *l*_*sup*_, *l*_*conf*_, *l*_*lift*_, *l*_*conv*_, and *h*_*conv*_ respectively.

8 $$ \text{count} (X) \equiv \sum_{S \in T} \left[ X \subseteq S^{*}\right]  $$

9 $$ \text{sup} (X) = \frac{{\text{count}(X)}}{|T|}  $$

10 $$ \text{sup} (a \rightarrow b) = \text{sup}(a \cup b)  $$

11 $$ \text{conf}(a \rightarrow b) = \frac{\text{sup}(a \rightarrow b)}{\text{sup}(a)}  $$

12 $$ \text{lift}(a \rightarrow b) = \frac{\text{sup}(a \rightarrow b)}{\text{sup}(a) \times \text{sup}(b)}  $$

13 $$ \text{conv}(a \rightarrow b) = \frac{1 - \text{sup}(b)}{1 - \text{conf}(a \rightarrow b)}  $$

The subset of candidate initiation rules for which these criteria met are called the set of *mined rules*. A rule is an element of the mined rules if and only if:

- count(*a*∪*b*)≥*l*_*count*_
- sup(*a*→*b*)≥*l*_*sup*_
- conf(*a*→*b*)≥*l*_*conf*_
- lift(*a*→*b*)≥*l*_*lift*_
- *l*_*conv*_≤conv(*a*→*b*)≤*h*_*conv*_

**Fig. 1:**
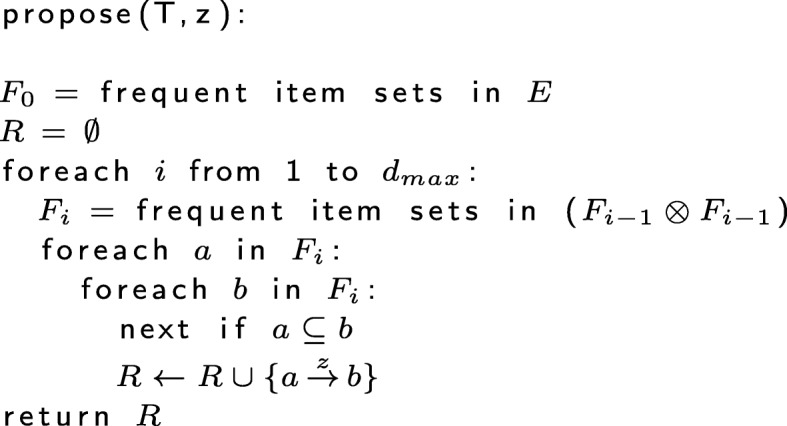
Rule proposal algorithm using the a priori principle

As with ARM, the a priori principle may be used with IRM to reduce the number of candidates that must be considered when testing a proposed initiation rule for inclusion. An item set *X* is considered frequent if a) count(*X*)≥*l*_*count*_ and b) sup(*X*)≥*l*_*sup*_. Let $d_{max} \in \mathbb {Z}^{+}$ be the maximum degree for which to mine rules. Thus, rule proposal can be implemented as shown in Fig. 1, where *X*⊗*Y* is the outer product of *X* and *Y*.

### Gateway rules

Recall that the gateway hypothesis predicts that the probability that *b* will arise out of *a* is greater than the probability that it would happen due to some other circumstance. When when we say this, we say that *a* is a gateway into *b* and denote that relationship using $a \rightsquigarrow b$.

An initiation rule is known as a gateway rule (denoted $a \rightsquigarrow b$) whenever the probability that *a*→*b* Eq. 14 is greater than the probability that any combination of the remaining antecedents will initiate *b*. This is equivalent to positing that *a* (either directly or indirectly) causes *b*.

14 $$ p(a \rightarrow b) = \frac{\sup(a \rightarrow b)}{\sup(b)}  $$

**Fig. 2:**
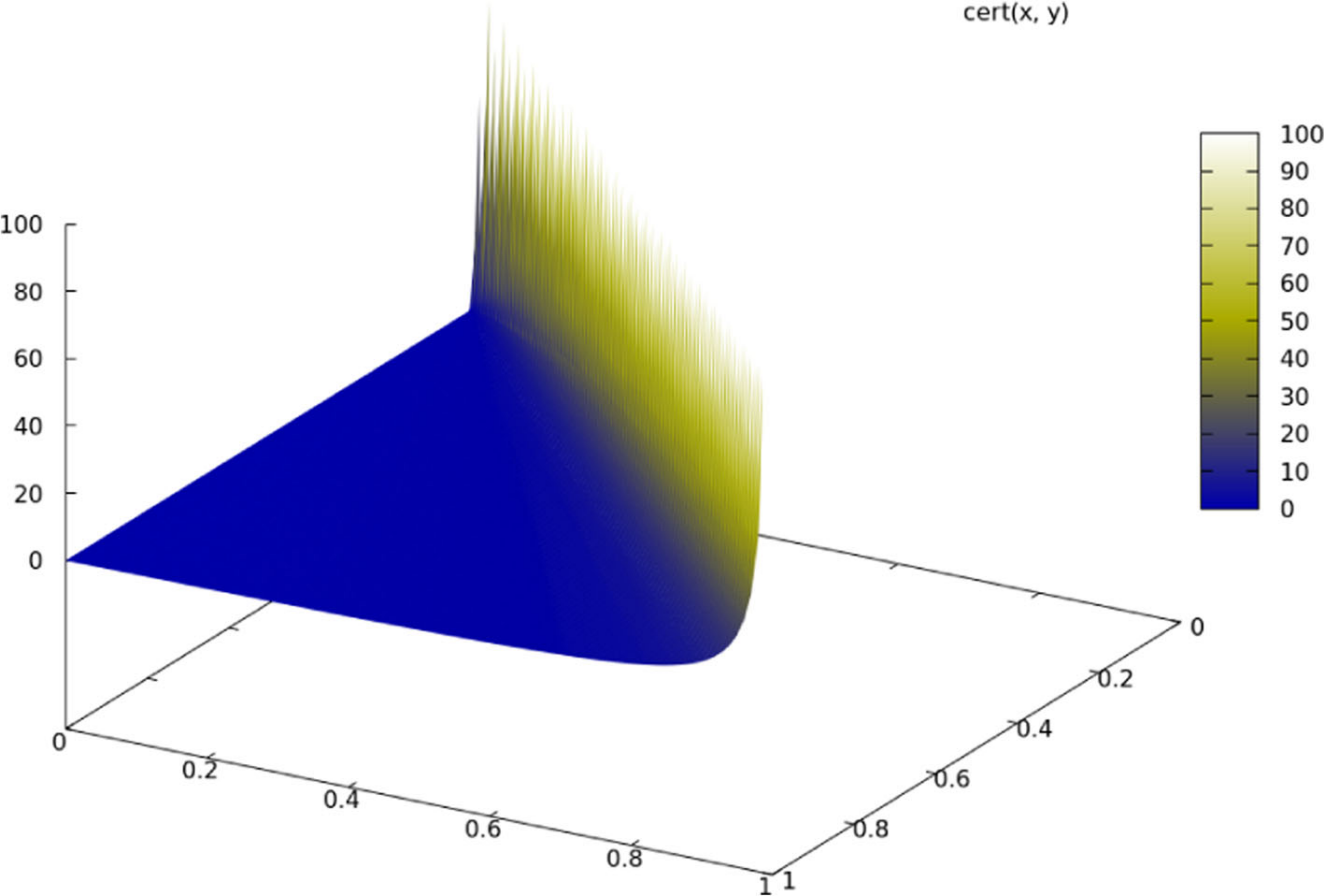
The domain of cert (*x*→*y*).Certainty approaches *∞* (i.e., becomes absolute) as sup (*x*→*y*) approaches sup (*y*). Color gradient represents certainty value for cert (*x*→*y*)

A simple way of ensuring this condition is to calculate the proposed rule’s *certainty* Eq. 1. The condition cert(*a*→*b*)=1 means that the probability that the subsequent arose out of *a* is precisely 50%, or alternatively that 50% of the remaining instances arose out of $a \nrightarrow b$. Therefore, by Eq. 1, we posit that *a* is the most likely cause of *b* when cert(*a*→*b*)>1. When the limit of *p*(*a*→*b*) approaches 1, cert *a*→*b* approaches *∞*: absolute certainty means that we posit *b* arises only from *a* Fig. 2

15 $$ \text{cert}(a \rightarrow b) = \frac{p(a \rightarrow b)}{1 - p(a \rightarrow b)} = \frac{\sup(a \rightarrow b)}{\sup(b) - \sup(a \rightarrow b)}  $$

This test is necessary (albeit not sufficient) for the assertion that a given event is the singular cause of another. Even in [1], this degree of causation is not predicted: cigarettes *or* alcohol leads to cannabis. This method could only be used to therefore test the idea that cannabis singularly leads to other illicit drugs.

To test the most general form of the gateway hypothesis, it must be the case that the association occurs by greater probability than chance alone. Thus, we suggest that that gateway rules can be established using the condition cert (*a*→*b*)>*l*_*cert*_, which is the maximum certainty for which we will reject *a*→*b* as causal. To satisfy this hypothesis, the *l*_*cert*_ must be at least the threshold where we would admit chance occurrence Eq. 16.

16 $$ l_{cert(*)} = \frac{1/|E|}{1 - 1/|E|} = \frac{1}{|E| - 1}  $$

Consider the transaction database in Table 1; if one calculates the count for all of the item sets and degree one rules in the transaction database, then the values provided in Table 2 can used to calculate support; for instance, *I*_1_ has a support of 0.8 because it is involved in 4 or 5 histories. Initiation rule support can be calculated by finding all histories where Eq. 4 holds; because of this, *I*_1_→*I*_3_ has a support of 0.6 (Table 2).

**Table 2:** Counts and supports for all item sets and unwindowed initiation rules of degree 1 in Table 1

I.S.	Count	Sup.	I.S.	Count	Sup.
*I*_1_	4	0.8	*I*_1_→*I*_2_	3	0.6
*I*_2_	5	1	*I*_1_→*I*_3_	3	0.6
*I*_3_	3	0.6	*I*_2_→*I*_3_	2	0.4
{*I*_1_,*I*_2_}	4	0.8	{*I*_2_,*I*_3_}	3	0.6
{*I*_1_,*I*_3_}	3	0.6	{*I*_1_,*I*_2_,*I*_3_}	3	0.6

Using this table, it is possible to derive the aforementioned metrics: for instance, lift (*I*_1_→*I*_3_)=(0.6)/(0.8×0.6)=1.25. To determine whether or not this *I*_1_→*I*_3_ is also a gateway rule, one calculates cert (*I*_1_→*I*_3_)=(0.6)/(0.6−0.6)=0.6/0. Although this value is undefined, it can be interpreted as approaching *∞*; thus, $I_{1} \rightsquigarrow I_{3}$ can be said to hold.

Likewise, cert (*I*_1_→1_2_)=(0.6)/(1−0.6)=1.5, so $I_{1} \rightsquigarrow I_{2}$ in an unwindowed context. Let us now form initiation rules over window *z*=2 (Table 3). Because the history for ID 3 does not initiate *I*_2_ within the window, the support for $I_{1} \overset {2}{\rightarrow } I_{2}$ drops to 0.4 and cert$(I_{1}\overset {2}{\rightarrow } 1_{2}) = (0.4)/(1 - 0.4) = 0.\overline {6}$. Thus, $I_{1} \rightsquigarrow I_{2}$ because the certainty of cert$(I_{1} \overset {2}{\rightarrow } I_{2}) \leq 1$ and therefore does not meet Eq. 1.

**Table 3:** Counts and supports for all item sets and initiation rules for *z*=2 and of degree 1 in Table 1

I.S.	Count	Sup.	I.S.	Count	Sup.
*I*_1_	4	0.8	$I_{1} \overset {2}{\rightarrow } I_{2}$	2	0.4
*I*_2_	5	1	$I_{1} \overset {2}{\rightarrow } I_{3}$	3	0.6
*I*_3_	3	0.6	$I_{2} \overset {2}{\rightarrow } I_{3}$	2	0.4
{*I*_1_,*I*_2_}	3	0.6	{*I*_2_,*I*_3_}	3	0.6
{*I*_1_,*I*_3_}	3	0.6	{*I*_1_,*I*_2_,*I*_3_}	3	0.6

### Visualization

The final phase that GatewayNet performs is visualization. This produces a directed graph which depicts relationships between initiation rules. Let *G* be a weighted digraph *G*=<*E*^′^,*R*>, where events *E*^′^ constitute the graph’s vertices, and rules *R* constitute edges between events. Let *r*∈*R* be a quadruple such that *r*≡<*e*_1_∈*E*^′^,*e*_2_∈*E*^′^,*w*,*c*>. Then for rule *a*→*b*, vertices {*a*,*b*}∈*E*^′^, edge *r*_*a*→*b*_ is defined by Eq. 17, and membership of *a*→*b* in *G* is defined by Eq. 18.

17 $$ r_{a \rightarrow b} = \left< a, b, \text{sup}(a \rightarrow b), \text{cert}(a \rightarrow b)\right>$$

18 $$ (a \rightarrow b) \in G \equiv \left< a, b,*,*\right>$$

### Synthetic data

To better characterize GatewayNet’s behavior, we created a synthetic data set (Additional file 2) for which interaction is well characterized. This data set is explicitly constructed so that a complete history is available for each subject in the data set.

The synthetic data was generated according to a mathematical model described in the following paragraphs. This was done for the purposes of validation; although we also tested against empirical data, it is important that we verify that GatewayNet is well-behaved. The forthcoming model describes a population which is fixated on events it considers preferential, but allows for experimentation with other events.

Let *E* consist of events {*I*_1_,*I*_2_,...,*I*_*n*_}, where $n \in \mathbb {Z}^{+}$, *E*_0_≡{*ε*}∪*E*, *e*_1_∈*E*_0_, and *e*_2_∈*E*_0_. A Markov chain of order 1 *P* (Additional file 1) is randomly constructed to represent transition probabilities from *e*_1_ to *e*_2_ (Table 4). Two real parameters are provided: the affinity *f* and interest *s*. Affinity represents the probability that a subject will be satisfied with *e*_1_ and will ensure that the event occurs at time *t*+1. Interest is a weight that represents the likelihood that the subject would independently ensure *e*_2_ will occur. A special event, *ε*, represents the *null event*, which represents a transition from no event.

**Table 4:** A sample Markov chain of order 1 describing transition probabilities between events {*ε*,*I*_1_,*I*_2_,*I*_3_}

	*ε*	*I*_1_	*I*_2_	*I*_3_
*ε*	0.750	0.050	0.120	0.080
*I*_1_	0.900	0.010	0.045	0.045
*I*_2_	0.250	0.500	0.550	0.160
*I*_3_	0.750	0.050	0.120	0.08

Naturally, each row in *P* must add to exactly 1.0; however, care must be taken to ensure that this criterion is met. Let *P*^′^ represent an |*E*_0_|×|*E*_0_| matrix. Each element of *P*^′^ is populated using Eq. 19: an event’s self-transition *e*_1_→*e*_1_ is simply represented by its affinity, while any other transition is randomly distributed from the remaining probability. Because the row sum may not add up to exactly 1, each element is then normalized across the row Eq. 20.

19 $$ P^{\prime}(e_{i} \rightarrow e_{j}) = \left\{\begin{array}{ll} a_{i} & i = j\\ \text{rand} \left[0, 1 - s_{j} P(e_{i} \rightarrow e_{j-1})\right] & i \not = j \end{array}\right.  $$

20 $$ P\left(e_{i} \rightarrow e_{j}\right) = \frac{P^{\prime}\left(e_{i} \rightarrow e_{j}\right)}{\sum^{|E_{0}|}_{k = 1} P^{\prime}\left(e_{i} \rightarrow e_{k}\right)}  $$

Because the generated history is considered to be a complete one, the initial state is always *ε*. Thus, time-point *t*=1 is considered to be the first opportunity for which an initiation event can occur. At each time-point, between two and three initiations may occur. Each history may have up to 12 records in it; in total, we generated 56,578 simulated transactions over 8192 histories. Most of the events had support above 10% (Table 5). In total, 29,412 events were generated, corresponding to an average of 2.49 events per history.

**Table 5:** Counts and supports for all 1-sets in the synthetic data

I.S.	Count	Sup.	I.S.	Count	Sup.
*I*_1_	7230	0.9117	*I*_7_	2891	0.3646
*I*_2_	2260	0.2850	*I*_8_	1508	0.1902
*I*_3_	7526	0.9491	*I*_9_	989	0.1247
*I*_4_	1304	0.1644	*I*_10_	1113	0.1404
*I*_5_	5632	0.7078	*I*_11_	661	0.0834
*I*_6_	3790	0.4763	*I*_12_	220	0.0277

**Fig. 3:**
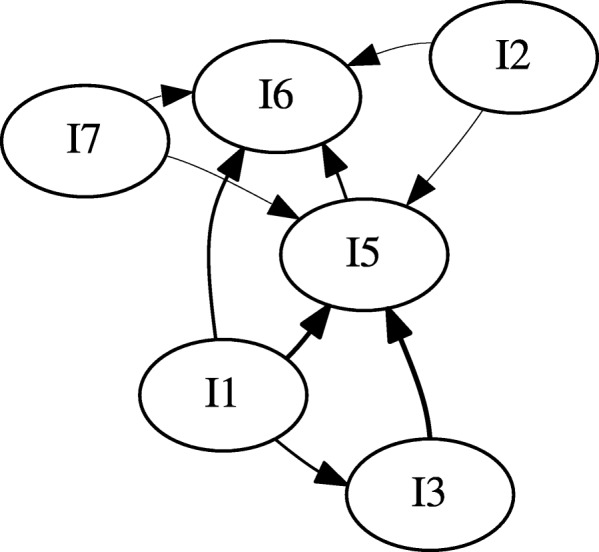
The synthetic data set expressed as a gateway network. This network is generated for *l*_*sup*_=0.05, *l*_*conf*_=0.5, and *l*_*lift*_=1.0. It is not apparent if any events qualify as gateway rules in this graph

**Fig. 4:**
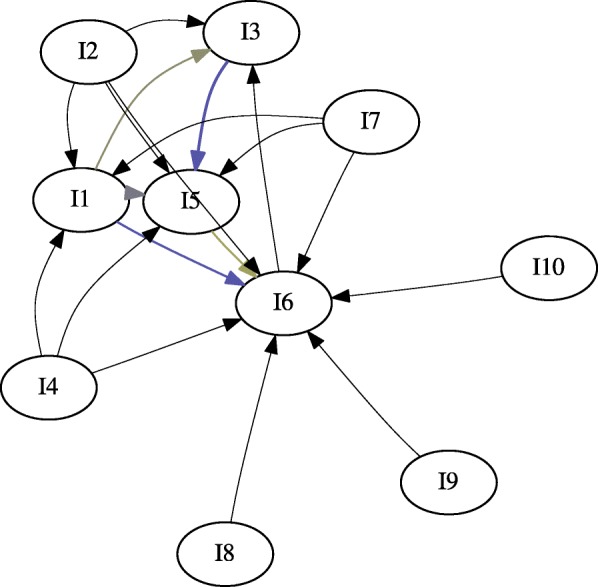
The gateway network for *l*_*sup*_=0.025. Expectedly, this network generates additional interactions. Color gradient represents the certainty of gateway rules

**Fig. 5:**
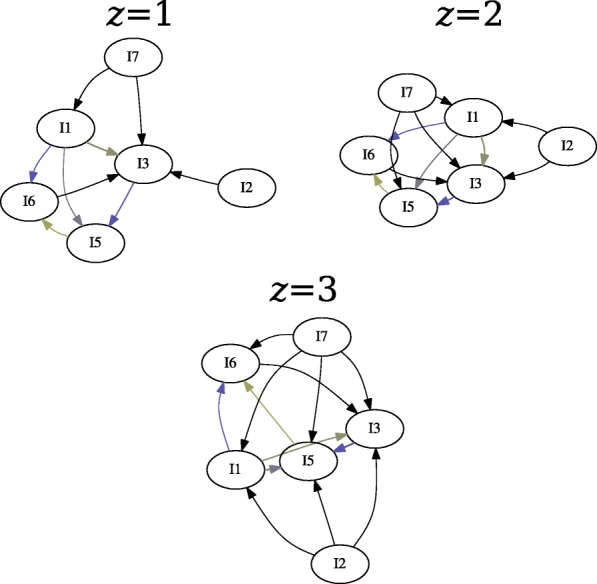
Windowed initiation rules for *z*∈{1,2,3}. In this particular case, windowing did not remove any vertices from the graph; however, additional edges are added as their support increases. Color gradient represents the certainty of gateway rules

We generated two gateway networks for the synthetic data: one for *l*_*sup*_=0.20 (Fig. 3), and one for *l*_*sup*_=0.025 (Fig. 4). In both instances, *l*_*conf*_=0.5, and *l*_*lift*_=1. Versions of the network without gateway rule highlighting, with gateway rule highlighting, and just the gateway rules were generated. Additionally, gateway networks were generated with window sizes of *z*=1, *z*=2, and *z*=3 (Fig. 5). This was done to determine whether windowing had an effect on the synthetic data.

### Empirical Data

Synthetic data is useful for evaluating the performance of GatewayNet since it is expected that some structures should arise within the output (therefore providing a method of validation). However, it should be noted that synthetic data does not necessarily model the real world; to test performance in that environment, an empirical data set was used.

UDS data obtained from 71,312 patient between August 1998 and June 2011 over nearly 111,359 emergency room visits at LSU Health Sciences Center (the hospital portion now belongs to University Health) in Shreveport, LA. This hospital is a Level I trauma center that serves the 7 parishes in LERN Region 7 (including the Shreveport/Bossier City area) [25]. Because Caddo Parish (where Shreveport resides) is adjacent to both the Texas and Arkansas borders, patients from east Texas and southern Arkansas are also frequently served.

During the screening interval, four screening panels were used, and during this time, some drugs were not tracked consistently. These drugs were: 3,4-methylenedioxymethamphetamine (MDMA or ecstasy) and methadone (tested during 2007–2011), methamphetamine and propoxyphene (1998–2000, 2002–2004), and barbiturates (1998–2007).

Prior to processing, we removed demographic data and then assigned each patient a random identifier (ID) by first shuffling the list of patients, then assigning each patient in the shuffled list a sequential ID. Additionally, screening dates were converted to their corresponding Lilian day number. The day number was then scaled by 1440 (the number of minutes in the day) and the time of screening in minutes was added to the date. Finally, each patient’s screening time was calibrated to the first by subtracting the first screening’s timestamp.

This was done for to ensure that the screening time is expressed as an integer. Additionally, because methamphetamine and MDMA are amphetamines and methadone is an opiate, any instance of either was converted to this category prior to any processing. Because many patients only visited once or did not test positive for any drugs, we restricted the list of histories to those with at least two time-points and at least one positive result. Finally, a history was only accepted if there was at least one more time-point following the time-point of the first positive result. In total, 11,364 histories over 42,745 time-points remained.

**Fig. 6:**
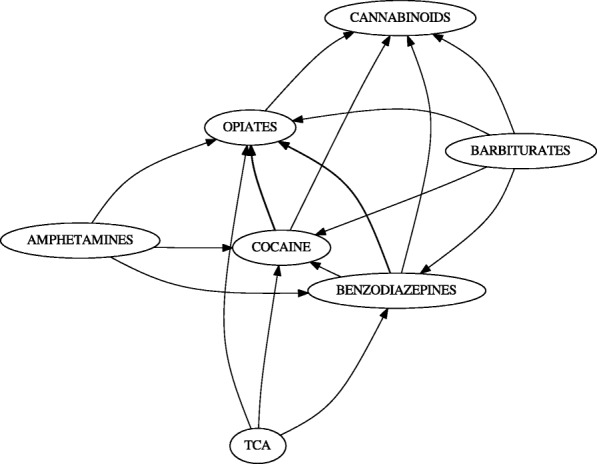
Gateway Network for LSUHSC-S data. No gateway rules were found; however, the initiations TCA → BENZODI- AZEPINES and BARBITURATES → BENZODIAZEPINES were discovered. Additionally, benzodiazepines, barbiturates, cocaine are seen to initiate cannabinoids

This data was first processed using unwindowed IRM (Fig. 6). We set the parameters *l*_*count*_=30, *l*_*sup*_=0, *l*_*conf*_=0.25. Minimum count was used instead of support because of the relatively few number of histories involving drug use (Table 6).

**Table 6:** Counts and supports for all 1-sets in the LSUHSC-S data

I.S.	Count	Sup.
Amphetamines	1459	0.1284
Barbiturates	666	0.0586
Benzodiazepines	4120	0.3625
Cannabinoids	5937	0.5224
Cocaine	3822	0.3363
Methadone	407	0.0358
Opiates	4525	0.3982
Phencyclidine	114	0.0100
TCAs	350	0.0308

**Fig. 7:**
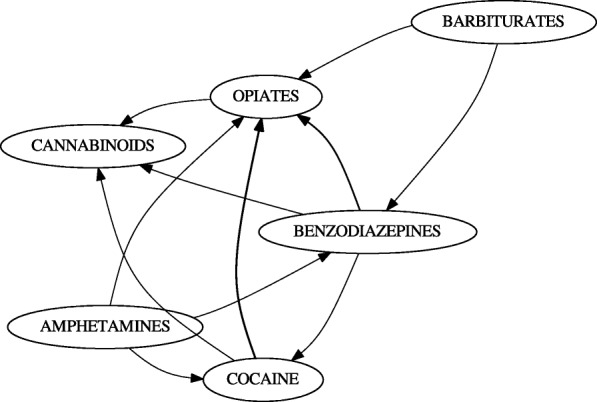
LSUHSC-S data over a year-long window. TCAs were eliminated when windowing was applied, and barbiturates no longer directly initiate cannabinoids

**Fig. 8:**
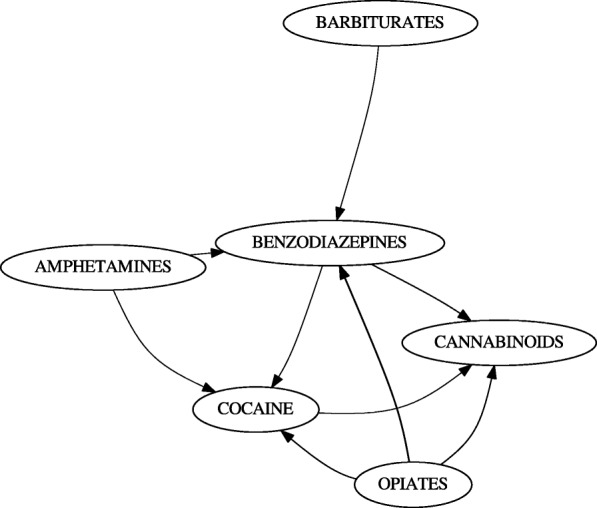
LSUHSC-S data with initiation of opiates removed. Rules with opiates in the antecedent were still retained. Cannabinoids remain the terminal initiation in this graph

In addition to performing unwindowed mining, we mined initiation rules within a window of 525,600 minutes (1 year) (Fig. 7). This was done to remove rules which were primarily supported by spurious positives. Opiates were sometimes administered to incoming patients or as a result of emergency surgery. Because of usage this arising from medical intervention rather than choice, we further removed rules of the form *x*→ OPIATES (Fig. 8).

## Results

Because we evaluated two data sets, we discuss the results for each data set separately. In the following subsection, we will discuss the results of applying GatewayNet to the synthetic data set. Afterwards, we discuss the results with respect to the empirical data from LSUHSC-S.

### Synthetic data

A relatively simple network with multiple interactions was generated (Fig. 3). It would appear that with our synthetic data set, event *I*_1_ initiates *I*_3_, *I*_5_ and *I*_6_, while *I*_5_ initiates *I*_6_ and *I*_3_ initiates *I*_5_. In this data set, *I*_1_, *I*_3_, and *I*_5_ are gateway events.

**Fig. 9:**
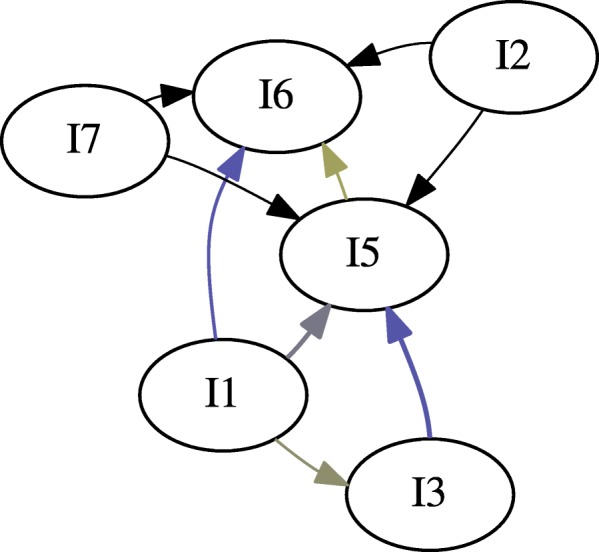
The gateway network with gateway rules highlighted. From this graph, we can tell that *I*_1_, 1_3_, and *I*_5_ are gateway events. Because of their blue color, *I*_1_ serves as a strong gateway into *I*_6_ and *I*_3_ into *I*_5_. Color gradient represents the certainty of gateway rules

**Fig. 10:**
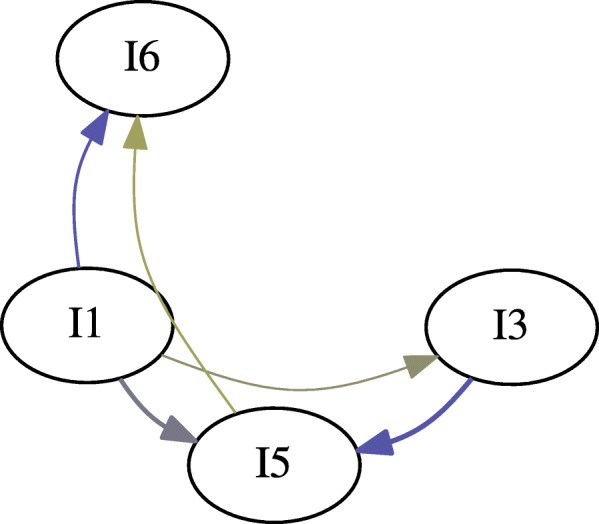
The gateway network with all non-gateway rules removed. Color gradient represents the certainty of gateway rules

Inspecting Fig. 3, there appear to be no clear gateway; however, this is misleading. The set of actual gateway events predicted by our algorithm are *I*_1_, *I*_3_, and *I*_5_, (Fig. 9). This may not be very surprising: these are also the most frequent events (Table 5). This can be more easily seen by removing the nodes which do not correspond to gateway rules (Fig. 10).

It can be clearly seen that $I_{3} \rightsquigarrow I_{5}$ with a high degree of certainty (32.3254). However, it is also true that $I_{3} \rightsquigarrow I_{5}$ (albeit with a weaker certainty of 19.4058) Fig. 5. Additionally, Both *I*_1_ (32.8393) and *I*_5_ (4.0736) are gateways into *I*_6_. The 2 ^*nd*^ degree rules {*I*_1_,*I*_3_}→*I*_5_ and {*I*_1_,*I*_5_}→*I*_6_ were mined.

In this case, windowing did not effect the vertices in the gateway network; however, the edges reported did change (Fig. 5). Notably, the number of edges associated with *I*_2_ and *I*_7_ increased with the window size. However, some of the rules associated with these events are not mined in the unwindowed sample, possibly due to diminishing support as maximum count increases.

### Empirical data

No gateway rules were observed in the data; however, some trends could be observed. In particular, tricyclic antidepressants (TCAs) and barbiturates both initiate benzodiazepine use (Fig. 6). This is not surprising; TCAs and barbiturates were once regularly prescribed, but have been replaced benzodiazepines (which have fewer risks). Although this does not qualify as a gateway event, it confirms that a known initiation even can be captured; the TCA relationship is also filtered out in the year-long window (Fig. 7).

Curiously, cocaine, opiates, and benzodiazepines initiate cannabinoids according to the data (Figs. 6, 7). In addition to barbiturates, amphetamines also initiate benzodiazepines (Figs. 6, 7). Before filtering out initiation rules involving opiates in the subsequent, cocaine, benzodiazepines, and amphetamines were also found to initiate opiates (Figs. 6, 7 and 8). The only drugs removed between the unwindowed and windowed variants were TCAs (Fig. 7).

## Discussion

As with the “Results” section, this section discusses the results for the synthetic data and empirical data separately. In the following subsection, we will discuss whether or not mining the synthetic data yielded the expected results. Afterwards, we discuss observations noted with respect to mining the LSUHSC-S UDS data.

### Synthetic data

The synthetic data showed a peculiar phenomenon: because *l*_*cert*_=1, it might be expected that one (and only one) event may serve as a gateway into another. As it turns out, this expectation is unwarranted: a high certainty means that *a* may be necessary to explain an event, not that it is sufficient to do so. In this case, two hidden gateway rules $\{I_{1},I_{3}\} \rightsquigarrow I_{5}$ and $\{I_{1},I_{5}\} \rightsquigarrow I_{6}$ were discovered. But what does this mean?

Let us first consider $\{I_{1},I_{3}\} \rightsquigarrow I_{5}$. One possibility is that *I*_1_ and *I*_3_ are co-requisite for the event. Consider the model from which the synthetic data is derived. In this model, an event for which there is high affinity will be quickly retained and will become recurrent; however, there is no reason that this might be the only event to occur within that time-point. Because of this, a history will show frequent experiments with other events: each event is an avenue for other events to occur alongside it.

With respect to drug use, this model is perhaps pessimistic: it predicts co-usage of one drug with other drugs, even when the user has high affinity with another one. In our model, frenetic experimentation seems to occur as the search for other events with high affinity continues. Is this a reasonable model of drug use? It is known that comorbidities in drug use often exist in reality. In Australasian countries, rates of experimentation of around 40% have been observed [11, 26].

If this does reflect trends in drug use, then it is an interesting result, as our method would be able to detect this phenomenon. In the synthetic data, *I*_1_→*I*_5_ at a probability of 95.099%, whereas *I*_3_→*I*_5_ with a probability of 96.999%. This concedes the possibility that they are used in combination.

But as it turns out, that is not the only possible explanation, because without any windowing, a gateway rule may be formed so long as *I*_1_ and *I*_2_ precede *I*_5_ at *any time in the past*. We should also note that $I_{1} \rightsquigarrow I_{2}$ with a probability of 94.069%. It possible that *I*_3_ is directly responsible for the transition into *I*_5_, and in reality, it is probably so: the first-order Markov chain used to model the relationships between events cannot explicitly express {*I*_1_,*I*_3_}→*I*_5_, nor does it actually encode *I*_1_→*I*_3_→*I*_5_ because it lacks the required history. Because of this, the latter is probably a better explanation.

### Empirical data

The LSUHSC-S data did not exhibit any gateway rules, and there are several potential reasons for this. It could simply be that the gateway hypothesis does not manifest itself in the population (or at least this sample). This represents acceptance of the null hypothesis that the drugs involved do not progress in development stages as predicted by Kandel and Yamaguchi [1–3].

However, it must be stated that there is another source of error which is likely to be present in the data: the drug screenings are collected during trauma center visits, and this offers an incomplete usage history of each patient. In many countries, illicit drug use is a criminal offense, and even if it were not, drug use is commonly voluntary and this means that intoxication is probably a desirable state for the user. Because of this, we can expect that patients will not seek the help of the trauma center merely because they have consumed an illicit drug; instead, we would expect to see that these patients will do so because of the perceived risk of dying or due to circumstantially related incidents.

Because of this, we do not know the patient’s drug history between intervention, and there may be biases due to the time it takes to clear metabolites from their systems. It is known, for instance, that some drug metabolites are excreted at different rates than others [27]. The cannabinoid metabolite 11-nor-9-carboxy- *Δ*9-tetrahydrocannabinol (THC-COOH) has a urinary half-life of about two days [28] compared to the 7.5 hour half-life of the cocaine metabolite benzoylecgonine [29]. This may mean that some initiation events will not be captured, particularly whenever screenings are separated by months or years.

Additionally, it was previously mentioned that all of the drugs tested by the panel (with the exception of cannabinoids) had a recognized medicinal use at the time. This true of opiates (for instance) to such a degree that initiations into opiates had to be filtered out of our data. However, we do not know if or when certain drugs were administered legitimately to patients (e.g., via prescription or surgical intervention prior to screening), and had gateway rules been found, they would have been suspect because of this.

We did, however, observe what is very likely to be this phenomenon in action. By finding that barbiturates and TCAs initiate benzodiazepines (Fig. 6), we were able to observe a known transition in medical practice. This initiation event was lost when initiation rules were limited to a year-long window (Fig. 7); since barbiturates and TCAs and benzodiazepines are antidepressants and anxiolytic respectively, it may have been the case that these were administered to patients en route as the result of psychiatric intervention. One may therefore predict that additional emergency intervention was simply not required within this window.

By removing rules with opiates in the rule’s subsequent, we notice that the transition into cannabinoids remains (Fig. 8). Interestingly, this is suggested by Kandel because she initially hypothesizes that the association is bidirectional [1]; in fact, we generally see this prediction also holding for benzodiazepines and cocaine. However, it must be restated that we did not find any gateway rules and that this is association. One potential explanation for this is that cannabinoids may have be easier to obtain illicitly over the sampling period than alternatives.

### Comparison to existing software

Several implementations of SRM are mentioned the “Discussion” section which may be compared to GatewayNet. As far as we are aware, no software utilizes the certainty measure, so any calculation thereof is extrinsic to other SRM software. However, a comparison can be made with existing software as long as support information of each item set mined is available.

To supply the other algorithms, we utilized SPMF, which implements both the SPADE and SPAM algorithms [30]. SPMF takes slightly different input than GatewayNet, and the input data was converted by assigning item labels to integer values and by combining each history into a single line. As SPMF does not have an option to set minimum count, this was achieved by using setting minimum support to 0.0005166 (30/58067 transactions). Both SPAM and SPADE produced equivalent output that varies only in output order and consists of 56,767,617 individual item sets. Because of this, the remaining analysis occurs on the SPAM output.

Unsurprisingly, both algorithms calculate the same number of 1-sets as GatewayNet does; however, special care must be taken to make the results comparable to GatewayNet’s because SPMF outputs its item sets as a series of time-points. In order to perform this comparison, we first “trimmed” SPMF’s item sets so that the repeated sequences at either extrema are truncated; then, each such item set with the maximum support is counted. In total, this reduced the number of item sets to 16,415,526.

**Fig. 11:**
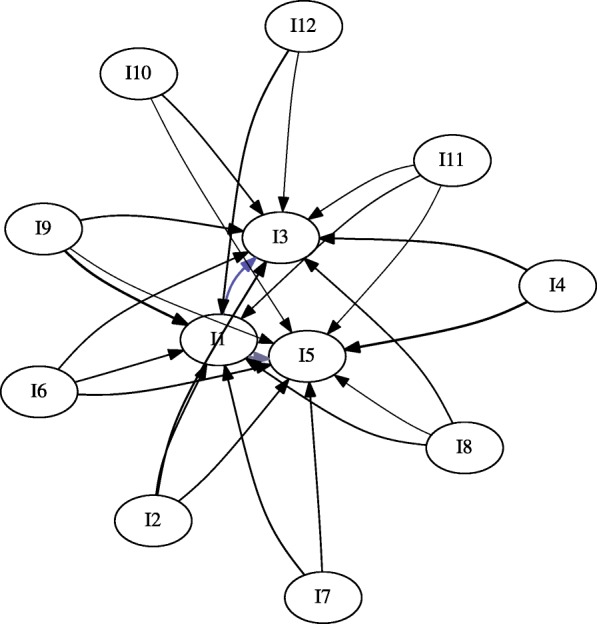
The gateway network in Fig. 6, except processed using SPMF. Two gateway rules, $I_{1} \rightsquigarrow I_{3}$ and $I_{1} \rightsquigarrow I_{5}$ remain highlighted, but other rules have dropped out because of incomplete item set counts

From this set, we calculated all frequent subsets in the data. This was first done by mining all subsets of length *k* for each SPMF item set *S*, for 0<*k*<|*S*|. A rules et was then generated with a minimum support of 0.025% and propagated to GatewayNet’s visualization software, gatewaynet-links. We found that no rule had a lift of 1 or greater; the graph has also reduced the set of gateway rules to $I_{1} \rightsquigarrow I_{3}$ and $I_{1} \rightsquigarrow I_{5}$ (Fig. 11). This might be explained by a loss of records due to SPMF’s output. In that output, {*A*,*B*} represents a single *transaction* where this set of events occurs, and it is distinct from {{*A*},...,{*B*},...} or {{*B*},...,{*A*},...}. In contrast, GatewayNet considers such histories to correspond to the item set {*A*,*B*}. Because of this, many small sequences of low support may be culled by the support threshold, therefore altering each item set’s frequency. Nonetheless, using SPMF as a basis for item set mining approaches the results of GatewayNet.

## Conclusions

IRM (as implemented by GatewayNet) shows promise to demystify the Gateway Hypothesis, but it may also be useful in the prediction of any event (as our synthetic set demonstrates). The quality of data provided to GatewayNet will strongly affect its output; however, with good data, it may not only be able to highlight initiation events, but also actual gateway events as well.

The ability of GatewayNet to predict initiation rules is expectedly dependent on data quality. In our case, emergency room UDS yielded no support for the gateway hypothesis; however, due to the nature of the screening, a full patient history might not be available.

It should be noted that GatewayNet is not presently optimized for speed. The a priori algorithm is known to be sub-optimal for association rule mining, and other SRM algorithms have explored optimization techniques regarding speed. In the future, we would like to explore techniques such as FP-growth as avenues for improving runtime speed.

## Availability and requirements

**Project name:** GatewayNet

**Project home page:** https://sun.cs.lsus.edu/software/gatewaynet/

**Operating systems:** GNU/Linux (amd64/x86_64)

**Programming language:** C++98/Perl

**Other Requirements:** Perl 5.22.1, File::Basename, Getopt::Long, GraphViz

**License:** Non-commercial Use

**Any restrictions to use by non-academics:** commercial use must be licensed

## Additional files

Additional file 1 Supplementary Data. This file contains the transaction database for our synthetic data. (TXT 702 kb)

Additional file 2 Supplementary Data. This file contains the Markov chain used to generate sample.txns.txt (CSV 3 kb)

## Abbreviations

ARM: Association rule mining
BBN: Bayesian Belief Network
ED: Emergency department
ID: Identifier
IRM: Initiation Rule Mining
LSUHSC-S: LSU Health Sciences Center in Shreveport
MDMA: Methylenedioxymethamphetamine
PCP: phencyclidine
SRM: Sequential Rule Mining
TCA: tricyclic antidepressant
THC: *Δ*9-tetrahydrocannabinol
THC-COOH: 11-nor-9-carboxy- *Δ*9-tetrahydrocannabinol
UDS: urine drug screening
